# Building coiled coils with polar cores

**DOI:** 10.1002/pro.70807

**Published:** 2026-09-16

**Authors:** Marcus D. Hartmann, Birte Hernandez Alvarez, Mikel Martinez‐Goikoetxea, Andrei N. Lupas

**Affiliations:** ^1^ Department of Protein Evolution Max Planck Institute for Biology Tübingen Tübingen Germany

**Keywords:** coiled coil, hydrophobic core, intrinsically disordered proteins, protein design, protein structure

## Abstract

Coiled coils are formed by α‐helices winding around each other into superhelical bundles. They are characterized by a specific geometry of interaction, called knobs‐into‐holes, in which residues in the core of the structure mesh regularly along a seam that runs the length of the helices. While these residues are predominantly hydrophobic, hydrophilic residues occur occasionally. In dimeric coiled coils, their sidechains are often sufficiently long to allow the head‐groups to extend out of the core and be solvated by water, but in trimeric and tetrameric coiled coils, they often cannot gain access to solvent and instead point inward, coordinating water molecules and ions along the central axis of the coiled coil. Building on this insight, we have used sequence motifs derived from trimeric autotransporter adhesins to design coiled‐coil sequences that lack hydrophobic sidechains for three or more consecutive heptads. Their crystal structures illustrate the strategies for accommodating extended stretches of hydrophilic residues within the coiled‐coil fold, which are confidently predicted as intrinsically disordered, raising questions about the actual structure of such proteins in their native environment.

## 1 INTRODUCTION

Coiled coils are helical bundles whose constituent α‐helices wind around each other in parallel or antiparallel orientation. Although their predominant form is of parallel dimers or trimers built on repetitions of an underlying seven‐residue sequence pattern, they can assemble into a great diversity of structures with different lengths, numbers of helices, sequence periodicities, helix crossing angles, degrees of supercoiling, and packing interactions (Hartmann, 2017; Lupas et al., 2017; Lupas & Bassler, 2017; Martinez‐Goikoetxea & Lupas, 2023). Because of this diversity, their classification into folds is awkward and inconsistent between different databases, such as SCOP (Chandonia et al., 2021), CATH (Waman et al., 2025), and ECOD (Schaeffer et al., 2025), all the more since they sometimes arise within other folds, or “grow” out of them through extension of helices constituent to those folds (Lupas & Gruber, 2005). Nevertheless, they have one important unifying feature, namely the packing geometry of their sidechains, which has been used to identify them in proteins of known structure (Kumar & Woolfson, 2021; Walshaw & Woolfson, 2001) and classify them by their architecture (Heal et al., 2018; Szczepaniak et al., 2021; Testa et al., 2009). This common geometry was first described by Francis Crick in his account of the coiled coil (Crick, 1952), in order to explain why the helices in the structure were supercoiled: “*So far, no reason has been given why the α‐helix should be deformed. It seems probable that this is due to the difficulty of fitting together the side‐chains of two adjacent α‐helices. […] If the side chains of the α‐helix are thought of schematically as knobs on the surface of a cylinder, then it is found that the pattern on this surface consists of knobs alternating with ‘holes’, that is, spaces into which the knobs of a neighbouring α‐helix could fit. The position of these holes is roughly independent of the exact nature of the surrounding side‐chains. […] It can be shown that by deforming the helices into coiled‐coils the knobs can be made to interlock systematically*.” The deformation needed to allow for a regular interlocking of sidechains results from the need to reduce the periodicity of an undistorted α‐helix from 3.63 residues per turn to 3.5 with respect to the packing interface, in order to allow the residues to occupy periodically recurrent positions. The seven positions of such a repeat are denoted a–g. Of these, two (a and d) point toward the center of the structure and form the knobs‐into‐holes interactions. Crick recognized that the energetics of these interactions would be governed by the hydrophobic effect (Crick, 1953): “*It is noticeable that the proportion of non‐polar to polar groups in tropomyosin is lower than for a more globular protein like haemoglobin. It is plausible to postulate that these non‐polar groups tend to occur between the chains, rather than on the outside of the molecule, since the packing of the hydrophobic groups together would allow more hydrogen bonds to be made elsewhere, and thus is energetically preferable. […] If this hypothesis were correct, one would expect there to be a tendency towards an alteration [sic] of polar and non‐polar residues in tropomyosin, the non‐polar occurring at an average interval of 3 1/2 residues, so that they always pointed ‘inside’ the molecule*.” This alternation of hydrophobic and hydrophilic residues within a seven‐residue periodicity is referred to as the heptad repeat.

The preference for hydrophobic residues at positions a and d of the heptad repeat is clearly apparent in most coiled coils, particularly if the pattern extends over hundreds of residues, such as in tropomyosin or myosin. However, hydrophilic residues do also occur at these positions. An early census of residue distributions in coiled coils found that in both a and d, about a quarter of residues were polar (Parry, 1982), a value that was confirmed by subsequent analyses (Gruber et al., 2006; Lupas et al., 1991). These overall tallies mask considerable variations across protein families. For example, the hyperthermostable stalks of surface proteins from crenarchaea, such as tetrabrachion (Peters et al., 1996), have only 10%–15% polar core residues, while the cytosolic coiled coils of eukaryotes, such as cortexillin (Faix et al., 1996) or EEA1 (Murray et al., 2016), typically have more than 30%. The overall tallies also mask family‐specific imbalances between positions a and d. Thus, in the coiled‐coil stalks of trimeric autotransporter adhesins (TAAs), which are long fibrous surface proteins of Gram‐negative bacteria (Bassler et al., 2015; Linke et al., 2006), about a quarter of core residues are polar, but only about 5% in position a, compared to 45% in position d (Hartmann et al., 2009). The same is true in reverse for the coiled coils of basic‐region leucine zipper proteins (Landschulz et al., 1988), where around 20% of core residues are polar, with 35% in position a (more than half due to the highly conserved asparagine in the third heptad; Lumb & Kim, 1995) and 5% in position d. Given this variation, as well as the existence of individual coiled coils with close to 50% polar core residues overall (such as the stalks of the Shiga toxigenic *Escherichia coli* [STEC] agglutination proteins of *E. coli*), we wondered whether these natural coiled coils are close to an upper limit for polar residues in the “hydrophobic” core, or it would in fact be possible to build a coiled coil that had 100% polar core residues, at least over extended parts of its sequence.

## 2 MATERIALS AND METHODS

### 2.1 Bioinformatic analyses

#### 2.1.1 Survey of the NxxQD motif in natural coiled coils

To assess the occurrence of the NxxQD motif in natural coiled coils, we searched the AlphaFold_UniProt100 database (version December 27, 2024) (Jumper et al., 2021) for protein sequences containing this motif. Approximately 4 million sequences (0.9% of the database) matched this criterion. We then filtered these sequences for motifs located within predicted coiled‐coil regions, which we defined as segments with an average COILS probability >50% over a 28‐residue window (Lupas 1996). This yielded ~120,000 sequences (~0.03% of the database; Table S3).

For the resulting dataset, we determined the predicted heptad register of the motif residues from the COILS predictions (Table S4), and tallied their frequencies. This analysis showed that within coiled‐coil proteins, NxxQD motifs occur predominantly outside the hydrophobic core, indicating that the motif is generally disfavored in coiled‐coil core positions.

#### 2.1.2 Structure prediction and computational analyses

To retrospectively analyze the structural propensities of our designs, we generated models using the AlphaFold3 webserver (Abramson et al., 2024). For the GCN4‐based constructs, we modeled variants containing single NxxQD motifs (GCN4‐NQD‐hep[2–4], Figure S1) and multiple motifs (GCN4‐NQD‐hep[23, 24, 34, 234], Figure S2), both in the presence and absence of chloride ions. For models including ions, we added one Cl^−^ ion per NxxQD motif. We evaluated model quality based on overall pLDDT values and agreement with our experimentally determined structures.

For the de novo designs, we generated models for sequence‐symmetrical of a designed coiled coil lacking hydrophobic residues (NHCC) and degenerate versions of a designed coiled coil lacking hydrophobic residues (degNHCC), with and without GCN4 adaptor sequences. As for the GCN4‐based constructs, we assessed the models using pLDDT scores and structural similarity to the corresponding crystal structures. Furthermore, to evaluate deviations in coiled‐coil geometry between the experimental and predicted structures (Figures S4 and S6), we calculated coiled‐coil structural parameters using samCC (Dunin‐Horkawicz & Lupas, 2010). We analyzed local conformational differences by calculating side‐chain χ1 dihedral angles for all residues (Figures S5 and S7). We predicted the intrinsic disorder propensities shown in Figures 4 and 6 with IUPRED3 (Erdős et al., 2021).

### 2.2 Experimental methods

#### 2.2.1 Cloning

To construct NHCC‐(GCN4‐pII)_2_ and degNHCC‐(GCN4‐pII)_2_, the corresponding DNA fragments were generated by primer‐extension PCR using the following oligonucleotide pairs:

nhcc P1 (5′‐GACCATGGTCTCCGATTGACGAAAACACCAAACGAACGGATGAGAATACTAAGCGCACTGACGAGAAC) with nhcc P2 (GACCATGGTCTCCTCATACGTTTCGTATTTTCATCCGTCCGCTTTGTGTTCTCGTCAGTGCGCTTAG), and degnhcc P1 (5′‐GACCATGGTCTCCGATTCAAGAAAACACCAAGCGCACCGACGAGAATACGAAACACACTCAGGAAACAAACAAGCGTACCGATC) with degnhcc P2 (5′‐GACCATGGTCTCCTCATCTGCTTTGTGTTCTCTTGAGTCTTTTGCTCCGTCTGATCGGTACGCTTGTTTGTTTCC). Amplified fragments were inserted into the pIBA‐GCN4tri‐His vector (Hernandez Alvarez et al., 2008) via the *Bsa*I restriction sites. The degNHCC‐(GCN4‐pII)_2hep_NC_ and degNHCC‐(GCN4‐pII)_2hep_C_ constructs were synthesized de novo (GenScript) and subcloned into pET‐30a(+) using *Nde*I and *Bam*HI sites. All recombinant clones were confirmed by sequencing.

#### 2.2.2 Protein expression and purification

All proteins were expressed and purified as described (Hernandez Alvarez et al., 2008) with minor modifications. Recombinant plasmids were transformed into *E. coli* TOP10 cells, which were cultured in LB medium at 37°C. At an OD600 of 0.5, protein expression was induced with either 0.2 μg/mL anhydrotetracycline for pIBA‐GCN4tri‐His‐derived constructs or 1 mM IPTG for pET‐30a(+)‐derived constructs. Cells were harvested 4 h post‐induction.

Bacterial cell pellets were resuspended in 20 mM Tris/HCl, pH 7.4, 50 mM NaCl, 5 mM MgCl_2_, and cOmplete Protease Inhibitor Cocktail (MERCK), 1 mM phenylmethylsulfonyl fluoride (PMSF), and 20 μg/mL DNaseI. Cells were broken using a French Press, and the lysate was stirred in denaturing buffer A (20 mM Tris/HCl, pH 8, 6M guanidinium hydrochloride, and 0.5M NaCl) for 1 h. The denatured lysate was centrifuged at 100,000 × *g* for 45 min and loaded onto a HisTrap FF column (Cytiva) equilibrated in buffer A. Bound His‐tagged proteins were eluted with a linear gradient of 0–0.5M imidazole in buffer A. The fractions containing the protein of interest were pooled, dialyzed against 20 mM Tris/HCl, pH 7.4, 150 mM NaCl, and concentrated using an Amicon Ultra Centrifugal Filter, 3 kDa MWCO (Millipore).

#### 2.2.3 Peptide synthesis

Peptides were commercially synthesized by GeneCust (France) and dissolved in 20 mM MOPS pH 7.5 and 50 mM NaCl at a concentration of 5 mg/mL. For each peptide, a second sample was prepared with an additional 5 mM CaCl_2_.

#### 2.2.4 CD spectroscopy

Circular dichroism (CD) spectroscopy was performed using a Jasco J‐815 spectropolarimeter equipped with a Peltier temperature controller. Proteins were prepared at a concentration of 20 μM in 10 mM Tris/HCl, pH 7.4, and 50 mM KF. Samples were loaded into a quartz cuvette with a path length of 0.1 cm and spectra were recorded from 190 to 240 nm at 0.1 nm intervals, with an average time of 1 s per point and a scan speed of 100 nm/min. Thermal melting experiments were performed by monitoring ellipticity at 222 nm while increasing the temperature from 10 to 100°C at a rate of 1°C/min. The melting temperature (*T*_m_) was determined by fitting the thermal denaturation curve using a sigmoidal Boltzmann function in the Spectra Manager software (JASCO). All measurements were repeated in triplicate, and buffer baselines were subtracted from sample spectra.

#### 2.2.5 Proteinase K digestion assay

Proteinase K digestion assays were conducted to assess protein stability and accessibility. Samples containing 5 μg of protein in 20 μL of digestion buffer (20 mM Tris/HCl, pH 7.4, 150 mM NaCl) were incubated with Proteinase K at a final concentration of 5 μg/mL at indicated temperatures. At the indicated time points, reactions were terminated by adding 0.5 μL of 100 mM PMSF. Digestion products were analyzed by SDS‐PAGE on 4%–20% Bis‐Tris gels (MERCK) in MES running buffer and visualized using Coomassie Brilliant Blue staining. Control reactions without protease were included to confirm specific digestion. Assays were performed in triplicate to ensure reproducibility.

#### 2.2.6 Crystallization, data collection, and structure solution

Crystallization was performed at 21°C via the vapor diffusion method. Initial trials were set up with a Honeybee 961 robot (Genomic Solutions Ltd.) by mixing 300–400 nL of protein solution and the same amount of reservoir solution on 96‐well sitting‐drop microplates, using commercially available screens. Best‐diffracting crystals were obtained under the following conditions: 0.05M zinc acetate, 20% PEG 3350 for NQD‐hep3 in the presence of 5 mM CaCl_2_; 0.2M sodium iodide, 20% PEG 3350, pH 7.0 for NQD‐hep3 in the absence of CaCl_2_; 0.1M sodium citrate pH 5.6, 35% *tert*‐butanol for NHCC; 0.1M sodium acetate pH 4.5, 3M sodium chloride for degNHCC; and 200 mM sodium chloride, 100 mM Bis‐Tris pH 5.5, 25% PEG 3350 for degNHCC‐(GCN4‐pII)_2hep_NC_. Prior to loop‐mounting and flash‐cooling in liquid nitrogen, selected crystals were transferred into droplets of reservoir solution supplemented with 30% glycerol (NHCC), 30% ethylene glycol (degNHCC), or 15% PEG 200 (degNHCC‐(GCN4‐pII)_2hep_NC_).

All data were collected at 100 K at the Swiss Light Source, using the beamlines, detectors, and wavelengths indicated in Table S2. Data were processed and scaled using XDS (Kabsch, 2010). Phasing of NQD‐hep3 in the absence of calcium was performed by single‐wavelength anomalous diffraction (SAD), exploiting the presence of ordered iodine ions. SHELXD (Sheldrick, 2008) was employed for heavy atom location as implemented in autoSHARP (Vonrhein et al., 2007), followed by substructure refinement using SHARP (de La Fortelle & Bricogne, 1997). After density modification with Solomon (Abrahams & Leslie, 1996), the ARP/WARP secondary structure recognition pipeline (Perrakis et al., 1999) could trace two helical segments in the asymmetric unit (ASU). All other structures were solved by molecular replacement in MOLREP (Vagin & Teplyakov, 2010) using the GCN4 structure 1GCN as a search model. All structures were completed through iterative rounds of manual model building in Coot (Emsley & Cowtan, 2004) and refinement with REFMAC5 (Murshudov et al., 1999), and all coordinates and structure factors deposited in the Protein Data Bank (PDB): NQD_hep3 with calcium (30LG), NQD_hep3 without calcium (30LH), NHCC (30LI), degNHCC (30LL) and degNHCC‐(GCN4‐pII)_2hep_NC_ (30YL). Data collection and refinement statistics are given together with the PDB accession codes in Table S2

## 3 RESULTS AND DISCUSSION

### 3.1 Constructs in a GCN4‐background—the NxxQD motif

Toward the goal of generating coiled coils with polar cores, we started with an approach that we had used successfully to study asparagine residues in position d (N@*d* [amino acid N at position *d* of the heptad repeat]), namely to introduce them sequentially into the GCN4 leucine zipper (Hartmann et al., 2009). Incidental to our analysis of the tail needle protein Gp26 of bacteriophage P22 (PDB: 2POH) for that study, we identified an intriguing polar core motif containing N@*a* and Q@*d* (NxxQD), which upon re‐refinement could be seen to coordinate a calcium ion (PDB:3C9I). We decided to explore this motif in a leucine zipper domain of *Saccharomyces cerevisiae* General Control Protein 4 (GCN4‐p1) background, constructing variants with one copy of the motif, either in the second or the third heptad, and with three consecutive copies, starting in the second heptad (Figures S1 and S2).

Peptides of the corresponding sequences were synthesized and CD spectroscopy was used to analyze the secondary structure of the three peptides in buffer with or without Ca^2+^. The single CD spectra revealed that the peptides are unstructured in solution up to a concentration of 150 μM, which was the highest concentration measured in this study. For structural analysis, the three peptides were set up for crystallization at a concentration of 1.5 mM (5 mg/mL). The peptide GCN4‐NxxQD‐hep3 carrying the motif in the third heptad (which naturally has an asparagine residue in position a) formed well‐diffracting crystals, both in the absence and in the presence of 5 mM CaCl_2_.

**FIGURE 1:**
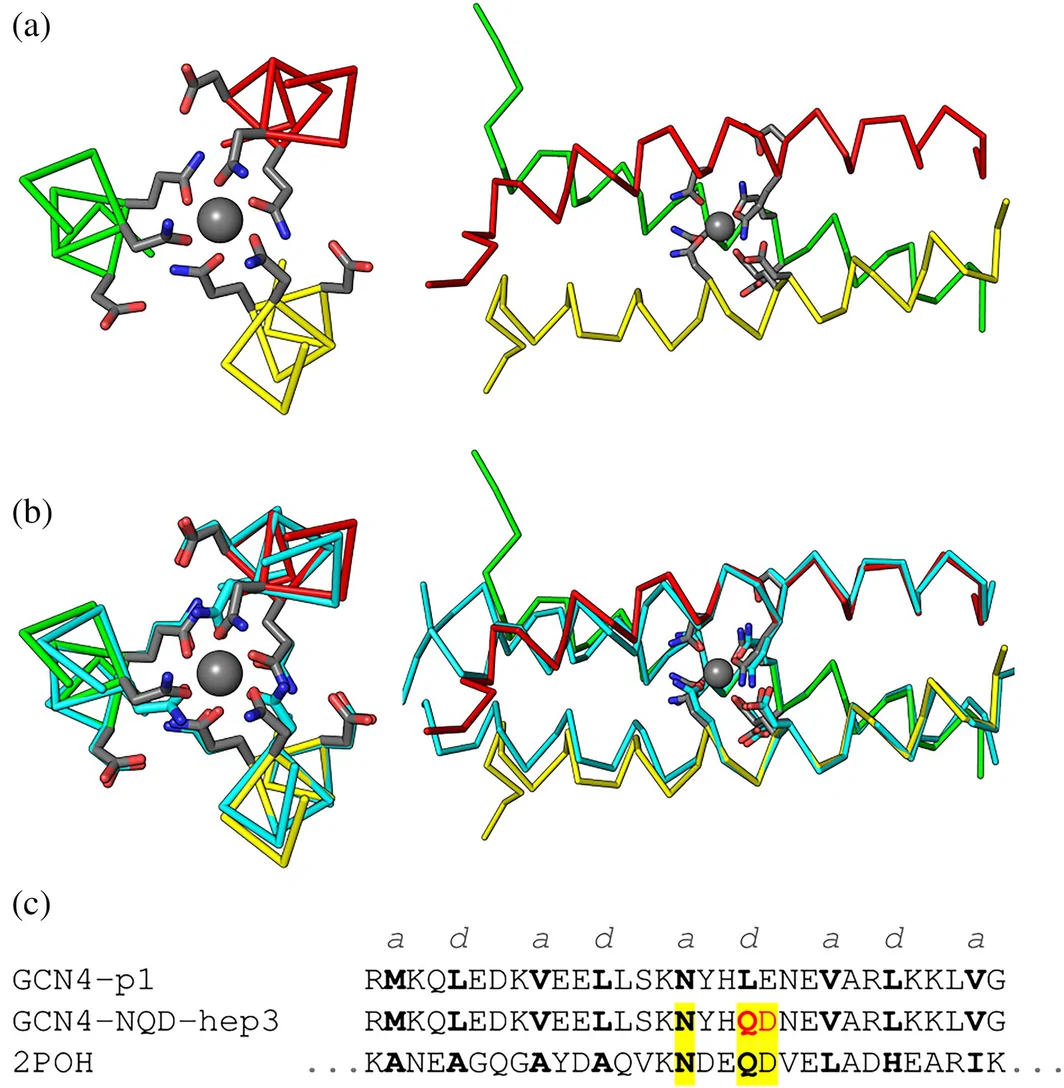
Comparison of our designed construct with a natural coiled coil featuring N@*d* hydrophilic core layers. (a) Top and side views of the NQD‐hep3 crystal structure, with N@*a* and Q@*d* coordinating a calcium ion (gray sphere). (b) Top and side views of the NQD‐hep3 crystal structure, superimposed to a segment of the tail needle protein Gp26 of bacteriophage P22 (Protein Data Bank: 2POH, in cyan). (c) Annotated sequence alignment of wild‐type leucine zipper domain of *Saccharomyces cerevisiae* General Control Protein 4 (GCN4‐p1) and the two structures. GCN4‐NQD‐hep3, the GCN4 leucine zipper with the motif N‐‐QD in heptad 3.

The form that crystallized in the presence of Ca^2+^ diffracted to a resolution of 1.6 Å and contained one chain in the ASU and was assembled into a trimer by crystallographic symmetry. Its structure is essentially identical to that of the corresponding region in the tail needle protein of phage P22, with a root‐mean‐square deviation (RMSD) in backbone atom positions of 0.89 Å over 21 residues centered on the NxxQD motif and an RMSD of 0.72 Å for all atoms of the motif itself (Figure 1). Here and in the tail needle protein, a central ion is coordinated in an octahedral geometry by the six oxygens of the N@*a* and Q@*d* sidechains, and the nitrogens of the Q@*d* sidechains are coordinated by the D@*e* residues. The central ion is well defined in the electron density, and its coordination distances and geometry, together with the presence of Ca^2+^ in the crystallization condition, allow a confident assignment as Ca^2+^.

**FIGURE 2:**
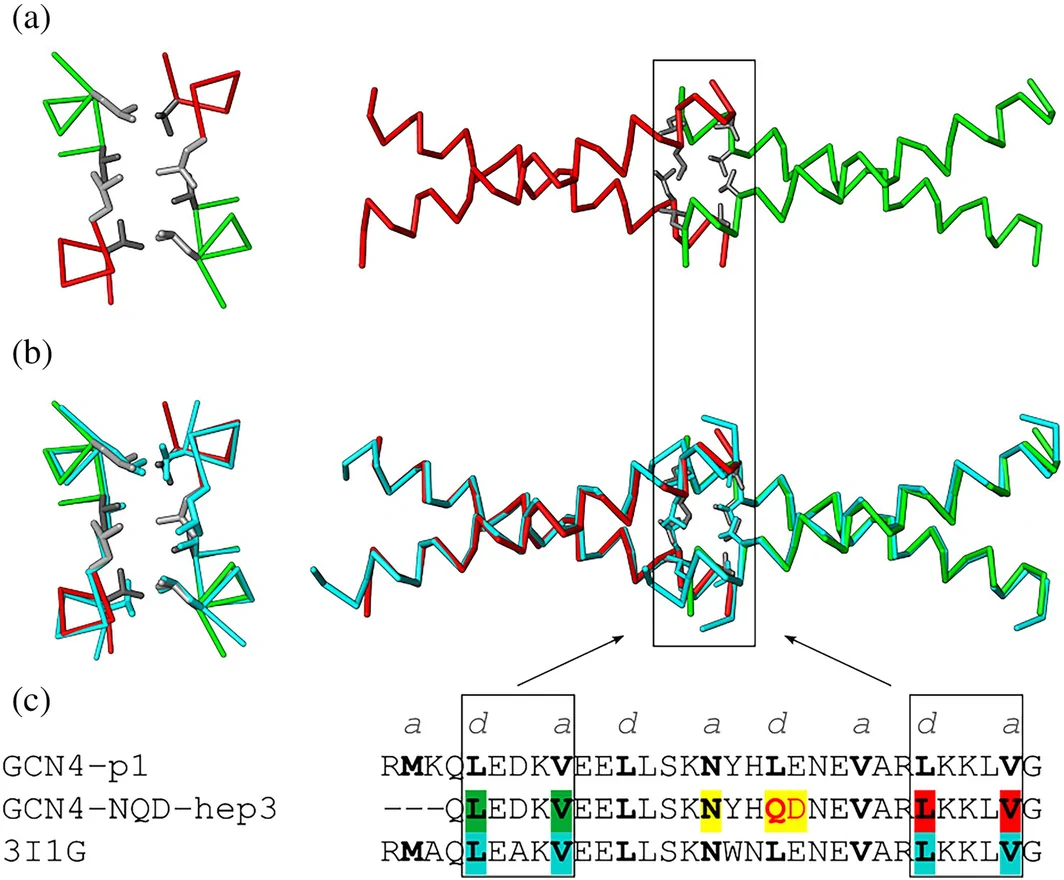
Comparison of our designed construct with another designed GCN4 variant featuring N@*d* hydrophilic core layers. (a) Top and side views of the NQD‐hep3 crystal structure in the absence of Ca^2+^. (b) Top and side views of a segment of a leucine zipper domain of *Saccharomyces cerevisiae* General Control Protein 4 (GCN4‐p1) variant (Protein Data Bank: 3I1G) superimposed to NQD‐hep3 (cyan). (c) Annotated sequence alignment of the two variants and the wild‐type GCN4. GCN4‐NQD‐hep3, the GCN4 leucine zipper with the motif N‐‐QD in heptad 3.

The form that crystallized in the absence of Ca^2+^ surprisingly did not form a coiled coil. Rather, two essentially straight helices associate at an angle of about 35° and assemble head‐to‐tail with other such dimers into long α‐helical rods. Assembly of dimers along the rod is mediated by two layers of hydrophobic core residues, Leu5/Leu26 and Val9/Val30, respectively (Figure 2a,c). In the crystal, the rods pack into roughly orthogonal arrays, with crossover points at Tyr17/His18 (the latter of which interacts with the free main‐chain carbonyl groups of a helix from the rod crossing over, providing a C‐cap). In all respects, this structure is nearly identical to that of another GCN4‐p1 variant reported previously (Diao, 2010), even though the two variants share no common mutations (Figure 2b,c).

All these constructs were conceived and tested experimentally in 2007, with much smaller protein sequence and structure databases, and a long time before the advent of AlphaFold. In an effort to understand retrospectively why none of our constructs was folded in solution and only one crystallized, we revisited them with up‐to‐date software and databases. As seen in Figures S1 and S2, AlphaFold3 models for the constructs show well‐folded, trimeric coiled coils with high confidence, with Ca^2+^ ions coordinated at the NxxQD motifs. In the one case for which we obtained a crystal structure (Figure 1), the RMSD between the C‐alpha atoms of model and structure is 1.7 Å. We also surveyed the occurrence of the NxxQD motif in natural coiled coils. For this, we filtered the UniProt database for protein sequences that featured an NxxQD motif within a predicted coiled‐coil domain (see Section 2). In the resulting dataset, the predicted coiled‐coil register of the NxxQD motif was, predominantly, out of the core, with N@*b*, N@*f*, and N@*c* accounting for 90% of the exemplars; the register of our constructs, N@*a*, corresponded to less than 4% of instances, indicating that this motif is unfavorable in the core (Table S4). AlphaFold models for these instances showed some cases with convincing geometry at high confidence, but a preponderance of poorly folded structures with low confidence, further supporting the disfavored nature of the motif in coiled‐coil cores. Retrospectively, this does not surprise us, since it corresponds to our starting assumption that polar residues in the core of coiled coils incur an energetic penalty, but the confident AlphaFold models for some trimeric coiled coils carrying the NxxQD motif, including our GCN4 variants, would nevertheless have encouraged us to test these constructs.

The failure to obtain stably structured GCN4‐p1 variants with the polar core motif NxxQD led us to try a de novo design in the next step.

### 3.2 A designed coiled coil with the four central heptads devoid of hydrophobic residues

Because of the inherent complexity of proteins with non‐redundant sequences, we decided to start our design effort with a coiled coil having perfect sequence symmetry (i.e., identical heptads). Our extensive experience with trimeric autotransporters (Bassler et al., 2015; Szczesny & Lupas, 2008), which are rich in coiled‐coil segments containing a high proportion of polar core residues, predisposed us to explore these for relevant sequence motifs. Some of the most extreme examples for polar cores in TAAs are shown in Figure S3:

(I) the putative adhesin BMI_I1862 of *Brucella microti* CCM 4915, in whose stalk of 40 heptads the central 37 contain Asn at position *d* (N@*d*); (II) the YadA‐like family protein of *Aggregatibacter segnis* ATCC 33393, with a stalk of 52 heptads, of which 49 consecutive ones contain a polar residue in position *d*, 39 having H@*d*; (III) the hemagglutinin‐domain protein Lferr_2308 of *Acidithiobacillus ferrooxidans* ATCC 53993, with a stalk comprising 51 heptads, of which 18 have a polar residue in *d* (16 consecutive ones N@*d*) and 16 have polar residues in both *a* and *d* (Q@*a*, D@*d*); and (IV) the autoagglutinating adhesin Saa of STEC (Paton et al., 2001), in whose coiled‐coil stalk half of all core positions are polar, including a recurrent motif with three consecutive positions carrying charged residues. Homologs of this adhesin, such as YadA‐like family proteins of *Pseudomonas aeruginosa* Pae‐32048 even have a preponderance of polar residues in the coiled‐coil core (57%), marking the highest proportion we have observed so far.

In search of an attractive starting point for the design of a coiled coil with an entirely polar core, we considered these four proteins. We decided against the stalks of the STEC adhesin because these are built by a repeat unit combining one 15‐residue segment (pentadecad) with one or two 11‐residue segments (hendecads), and we wanted to remain within the canonical structure space of heptads. We also decided against the stalk of Lferr_2308 because its polar heptads never occur in consecutive repeats and are always flanked by at least three canonical core layers built of Ile or Leu. In the end, we opted to start with by far the most common polar core residue in TAAs, N@*d*, which is also the dominant motif in the stalk of BMI_I1862. This residue is followed in about half the cases by T@*e* (Hartmann et al., 2009). Into this starting pattern, ‐‐‐NT‐‐, we inserted a K → E(+4) intrahelical interaction in order to *improve* helicity (Baker et al., 2015; Marqusee & Baldwin, 1987), obtaining ‐‐ENTK‐. Next, we looked for a motif to stabilize the trimeric structure. A frequently encountered motif forming inter‐helical salt bridges in TAAs is R@*g–*D@*b*, bracketing position *a* of the heptad repeat (Alvarez et al., 2010); we therefore included this to obtain ‐DENTKR. Finally, we opted for T@*a*; this is the second most frequent polar residue in position *a* of trimeric coiled coils after Gln, as judged from the CC+ database (Testa et al., 2009), but offers a methyl group in its side‐chain, which we felt could be useful as a minimal hydrophobic point of contact. We refer to constructs carrying repeated instances of TDENTKR as NHCC, for non‐hydrophobic coiled coil.

Our first construct, comprising five TDENTKR repeats, was unstructured in solution by CD spectroscopy under all conditions tested and did not crystallize. We therefore continued our efforts with a construct in which four repeats of TDENTKR were fused to the trimeric variant of the GCN4 leucine zipper, GCN4‐pII, at both the N‐ and C‐termini to yield NHCC‐(GCN4‐pII)_2_ (Table S1). We had established previously that using GCN4 adaptors is an effective strategy to support the folding and stability of oligomeric coiled‐coil fragments (Deiss et al., 2014; Hartmann et al., 2012; Hernandez Alvarez et al., 2008) and we hoped that in a shorter construct, these stabilizing effects would be more pronounced.

**FIGURE 3:**
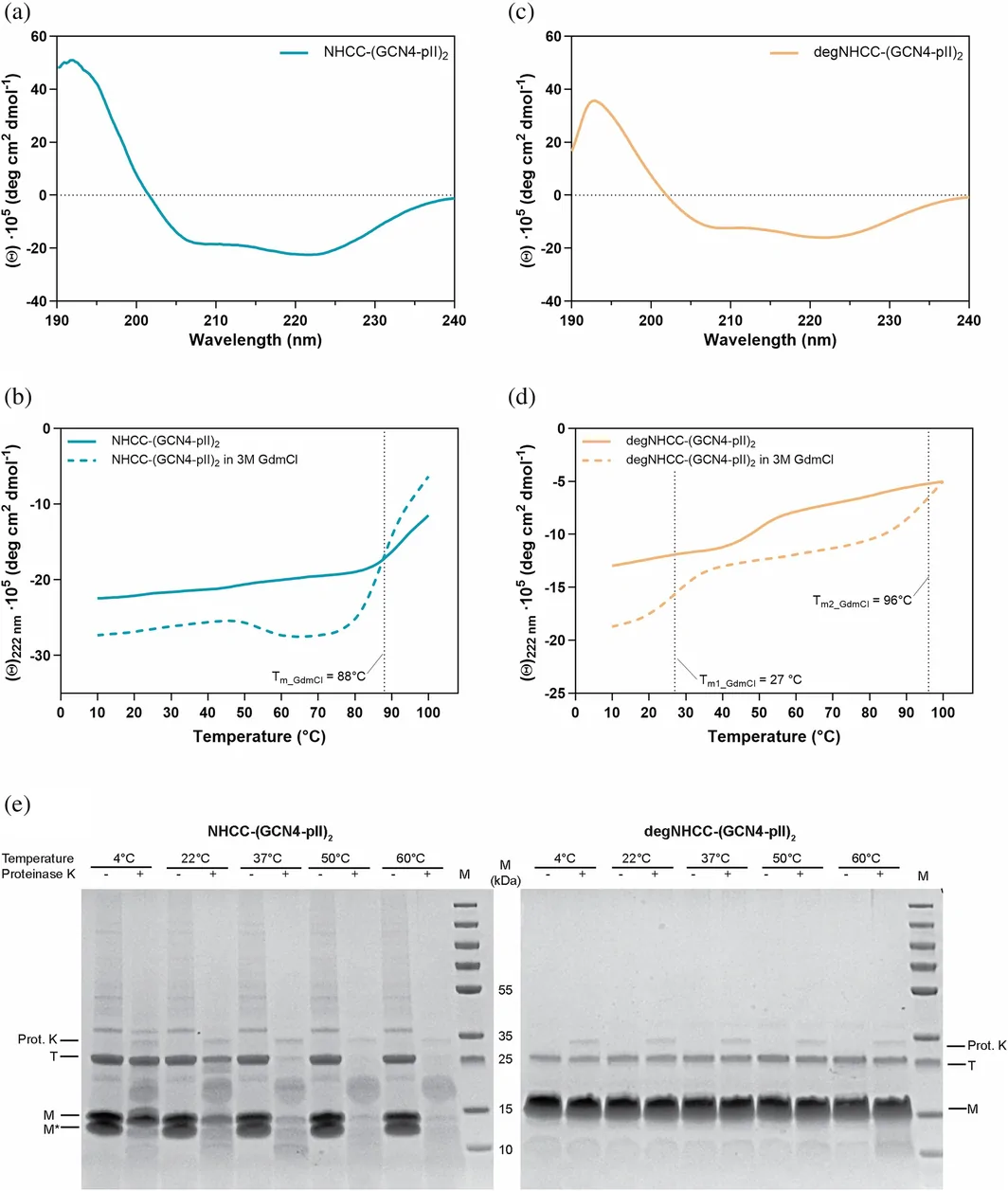
Stability analysis of NHCC‐(GCN4‐pII)_2_ and degNHCC‐(GCN4‐pII)_2_. (a) Circular dichroism (CD) spectrum of NHCC‐(GCN4‐pII)_2_. (b) Thermal melting curve of NHCC‐(GCN4‐pII)_2_ monitored by ellipticity at 222 nm. (c) CD spectrum of degNHCC‐(GCN4‐pII)_2_, introduced in a subsequent section. (d) Thermal melting curve of degNHCC‐(GCN4‐pII)_2_ monitored by ellipticity at 222 nm. (e) Proteinase K digestion assay for NHCC‐(GCN4‐pII)_2_ and degNHCC‐(GCN4‐pII)_2_ at increasing temperatures. degNHCC, degenerate versions of a designed coiled coil lacking hydrophobic residues; NHCC, sequence‐symmetrical of a designed coiled coil lacking hydrophobic residues.

Analysis of the NHCC‐(GCN4‐pII)_2_ construct by CD spectroscopy revealed a characteristic α‐helical spectrum with a maximum at 192 nm and typical minima at 208 and 222 nm (Figure 3a). The [Θ]_222nm_/[Θ]_208 nm_ ellipticity ratio was 1.2, which is indicative of a coiled‐coil arrangement of the α‐helices (Greenfield, 2006). Upon heating, NHCC‐(GCN4‐pII)_2_ unfolds in a single step with melting temperatures of *T*_m_ = 95°C in buffer and *T*_m_GdmCl_ = 88°C in 3M guanidinium hydrochloride (Figure 3b). The *T*_m_ values obtained in this study correspond to previously reported values for a GCN4‐pII peptide (Harbury et al., 1993). We conclude that these melting curves most likely show the unfolding of the terminal GCN4‐pII adaptors, and that the four central TDENTKR repeats are of low stability, a conclusion that is further substantiated by the high susceptibility of the NHCC‐(GCN4‐pII)_2_ construct to Proteinase K already at low temperatures (Figure 3e). Nevertheless, NHCC‐(GCN4‐pII)_2_ crystallized readily and its structure could be determined to a resolution of 1.9 Å.

The NHCC structure showed that the success of an important design goal, the introduction of intra‐ and inter‐helical salt bridges, is difficult to estimate, as many of the sidechains outside the interfaces of the helical bundle could not be resolved in the crystal structure. Thus, of the nine designed RxD interchain motifs, three formed salt bridges in the crystal structure, two did not, and four could not be resolved. Of the 12 designed ExxK intrachain motifs, 1 formed a salt bridge, 5 did not, and 6 could not be resolved.

**FIGURE 4:**
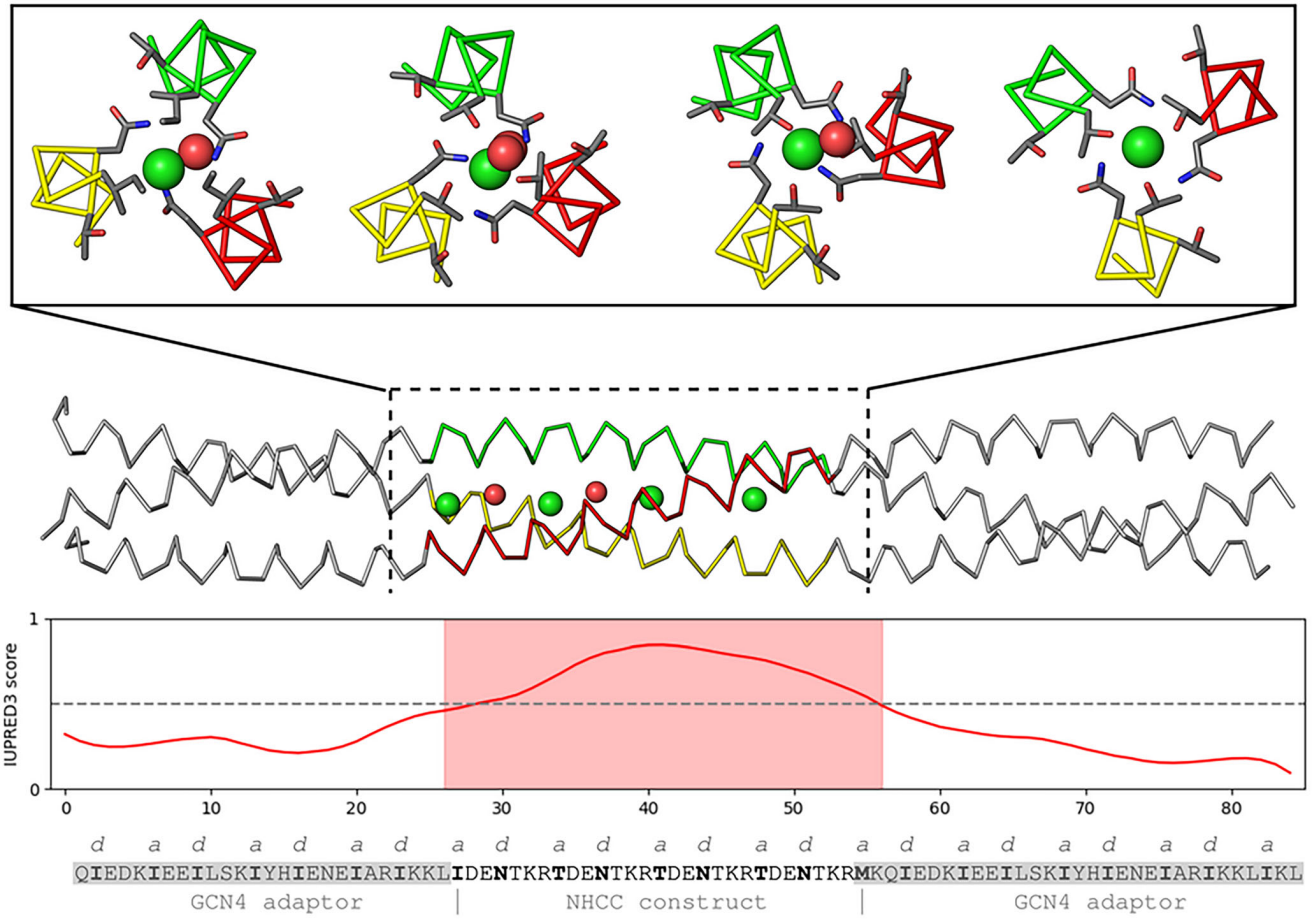
Detail of the a/d/e layers in the crystal structure of our sequence‐symmetrical of a designed coiled coil lacking hydrophobic residues (NHCC) design. (Top) Cross section of the four TDENTKR repeats and lateral view of NHCC; the design could only be crystallized when expressed with N‐ and C‐terminal GCN4 adaptors (gray). The side‐chains of the *a*/*d*/*e* layers are shown as sticks, and chloride ions are shown as green spheres. Water molecules are shown as red spheres. (Bottom) IUPRED3 disorder prediction, with the segment above 0.5 highlighted in red, and annotated sequence of NHCC.

A surprising aspect of the NHCC structure was the fact that, although the coiled coil is—as designed—a homotrimer, it displays asymmetric features, particularly in its side‐chain conformations (Figure 4). This is most conspicuous in the N@*d* layers, which normally form a highly ordered network of polar interactions with an anion at its center. In our 1.9 Å structure, each N@*d* layer indeed coordinates a chloride ion at its center, which could be confidently assigned based on its well‐defined electron density, coordination geometry, and coordination distances. However, the network of polar interactions is disrupted by the infiltration of water molecules, affecting the core in all but the most C‐terminal layer. The water molecules are partly coordinated by the T@*a* layers, which point their hydroxyl groups toward the center of the bundle, not the solvent, and thus do not provide the anticipated minimal hydrophobic point of contact. It is unclear whether the infiltration of water is the driver of the asymmetry, or if the intrinsic asymmetry of the structure is stabilized by the water molecules. In the context of the biophysical measurements and the protease sensitivity of NHCC, even in fusion with GCN4 adaptors, we interpret the asymmetry as indicating a lack of structural specificity of our design. We therefore think that the TDENTKR repeats in our construct exhibit dynamic behavior and sample multiple conformations in solution, some of which can be immobilized sufficiently by coordination of water molecules to allow crystals to form.

As for the NxxQD constructs, the NHCC design was conceived and experimentally validated prior to the advent of AlphaFold, which prompted us to do a retrospective analysis. Although the AlphaFold models for NHCC lacking GCN4 adaptors showed mostly low‐confidence helical bundles, including the adaptors produced models that closely matched the experimental structure (RMSD <1 Å, pLDDT >90). Notably, the sidechains of the T@*a* layers in the AlphaFold models adopted the correct, though unexpected geometry, with their hydroxyl groups oriented toward the bundle core, rather than the solvent. These models did not feature the asymmetries in the N@*d* layers that we observed in the crystal structure (Figure S5). The partly unconvincing models produced by AlphaFold are probably related to the low intrinsic stability of the TDENTKR repeats, as also seen in the IUPRED3 disorder prediction scores (Figure 4).

As we were not able to obtain folded material from the NHCC construct without GCN4 adaptors, we revised the design toward resembling more closely a natural coiled coil.

### 3.3 An attempt to mimic natural sequences through loss of sequence symmetry

As judged by the asymmetric features and susceptibility to proteolysis in a proteinase K digestion assay, NHCC lacks structural specificity and exhibits dynamic behavior, which allows it to sample alternative local conformations, even when fused to GCN4 adaptors. We reasoned that the perfect sequence symmetry of the design may have contributed to this behavior, since any unfavorable interactions in the sequence would be amplified along the length of the construct. Natural coiled coils rarely feature perfect sequence repeats even locally, providing a way to introduce compensatory interactions that increase structural specificity and reduce conformational dynamics. An interesting phenomenon related to this aspect concerns the folding of coiled coils in the correct register, given that repeating heptad sequence patterns may have many alternative ways to associate that are largely isoenergetic. In natural coiled coils, this issue appears to have led to sequences that are close to the unstructured state (Gáspári & Nyitray, 2011; Lupas et al., 2017), except for a local motif that is stably helical and has the propensity to oligomerize. This “trigger” motif serves as a nucleation site for the folding of the coiled coil and ensures that this proceeds in the correct register (Kammerer et al., 1998; Steinmetz et al., 1998).

**FIGURE 5:**
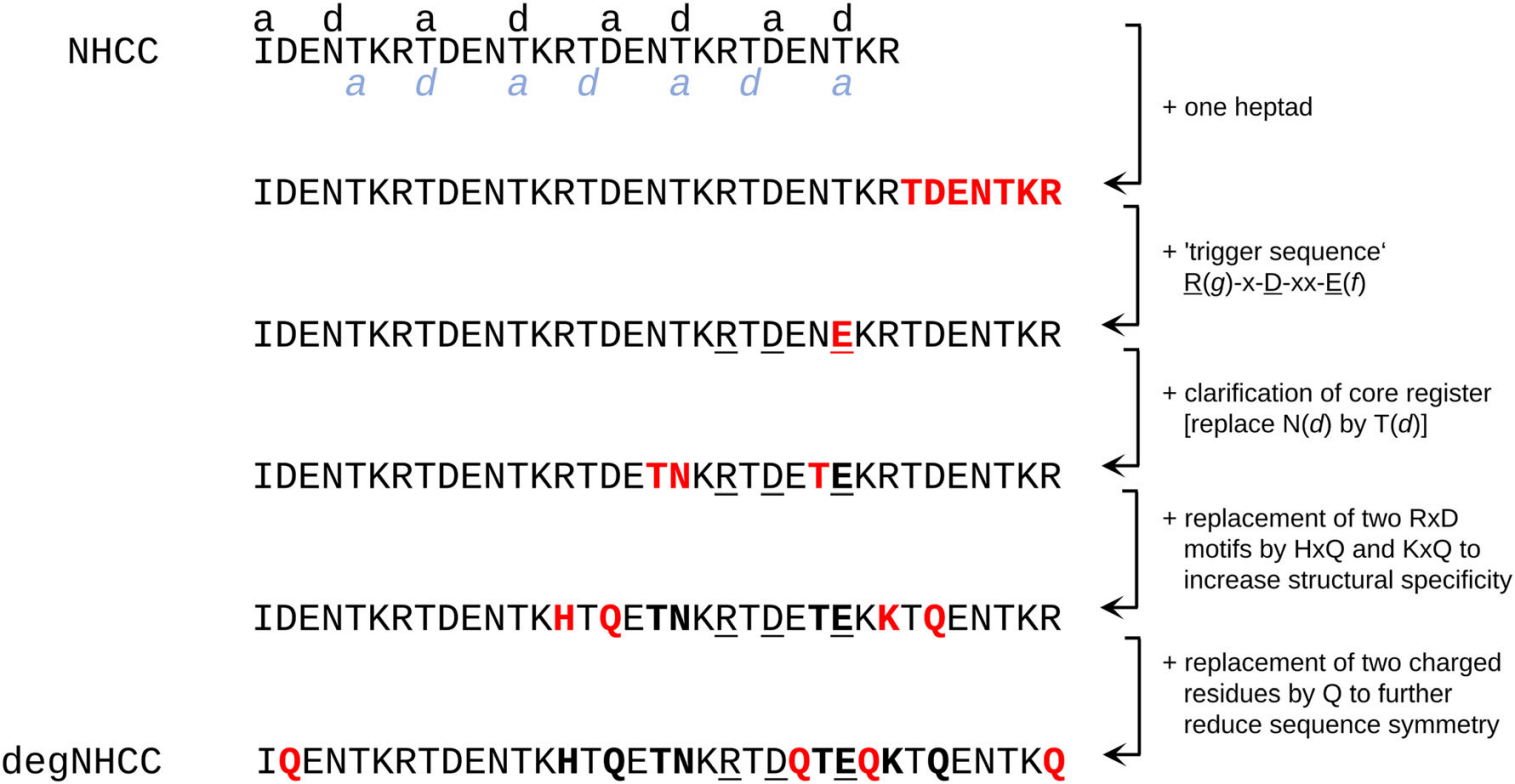
Schematic diagram of the rationale behind the degenerate versions of a designed coiled coil lacking hydrophobic residues (degNHCC) design. NHCC, sequence‐symmetrical of a designed coiled coil lacking hydrophobic residues.

In an effort to improve the structural specificity and stability of the NHCC design, we introduced a series of changes to better mimic features typical of natural coiled coils. The steps are detailed in Figure 5. We restored the length of the construct to five heptads, which we had reduced to 4 when we added the GCN4 adaptors. We converted one of the R(g)‐x‐D motifs to a canonical trigger motif, R(g)‐x‐D‐xx‐E(f) (Bjelić et al., 2013). We changed threonine residues in the recurrent N(d)‐T motifs, which could potentially have specified an alternative core register (gray in Figure 4), in order to clarify the correct frame. We removed two instances of the R(g)‐x‐D motif to further emphasize the newly introduced trigger sequence, replacing them with HxQ and KxQ, respectively. Finally, we alleviated the sequence repetition further by replacing two charged residues with Q. This yielded the final design, which we named degNHCC for degenerate NHCC.

Like NHCC, degNHCC was unstructured in solution and failed to crystallize, so in a next step, we fused it to GCN4‐pII adaptors at both termini. The CD spectrum of degNHCC‐(GCN4‐pII)_2_ showed a characteristic α‐helical profile with a prominent maximum at 193 nm and pronounced minima at 208 and 222 nm (Figure 3c). The [Θ]_222nm_/[Θ]_208 nm_ ellipticity ratio, which is >1, is indicative of a coiled‐coil structure. Unlike NHCC‐(GCN4‐pII)_2_, the thermal melting curve of degNHCC‐(GCN4‐pII)_2_ exhibited a single unfolding transition in buffer (*T*_m_ = 53°C) and two transitions in 3M guanidinium chloride (*T*_m1_GdmCl_ = 27°C, *T*_m2_GdmCl_ = 96°C), suggesting a sequential unfolding process, in which degNHCC heptads denature first, followed by the GCN4‐pII adaptors. This is further substantiated by the protease assay showing that degNHCC‐(GCN4‐pII)_2_ resists Proteinase K digestion up to 50°C. We conclude that the redesign of NHCC to degNHCC indeed improved both the structural specificity and stability of the construct, which led to well‐diffracting crystals and allowed us to determine a structure at a resolution of 1.84 Å.

**FIGURE 6:**
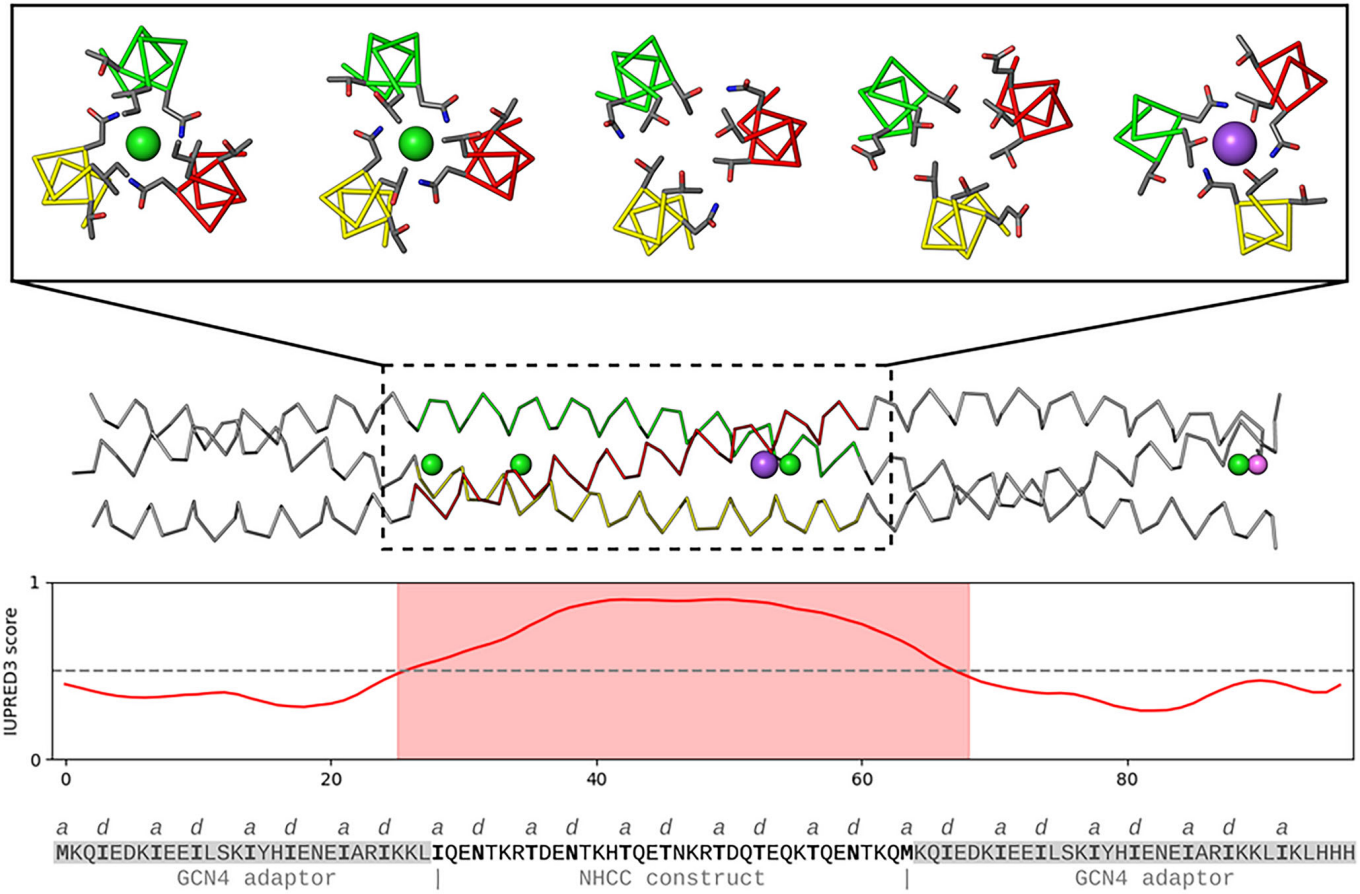
Detail of the a/d/e layers in the crystal structure of our degenerate versions of a designed coiled coil lacking hydrophobic residues (degNHCC) design. (Top) Cross section of the five designed heptads and lateral view of the structure; the design could only be crystallized when expressed between GCN4 adaptors (gray). The side‐chains of the a/d/e layers are shown as sticks; chloride (green), sodium (purple) and nickel (pink) ions are shown as spheres. (Bottom) IUPRED3 disorder prediction, with the segment above 0.5 highlighted in red, and annotated sequence of degNHCC. NHCC, sequence‐symmetrical of a designed coiled coil lacking hydrophobic residues.

Like the NHCC design on which it was based, degNHCC folded into a trimeric coiled coil. Unlike the fully‐repetitive design, however, it did not display any asymmetries in its side‐chain conformations, nor did the electron density suggest infiltration of water molecules into the core, albeit we identified three different types of ions in the structure, according to their coordination geometries and electron densities (Figure 6). Like in NHCC, the three N(d)T motifs that remained after redesign coordinate a chloride anion at their center; additionally, one of these chloride ions is in a complex with a sodium ion, which is itself coordinated by the preceding T@*a* layer. Generally, the T@*a* layers point their hydroxyl group toward the center of the bundle, except, unexpectedly, for the one in the second heptad (Thr36), which orients it toward the solvent, being partially coordinated by the preceding arginine (Arg35). There are two more ions localized in the center of the coiled‐coil bundle, albeit outside the designed heptads. The C‐terminal His‐tag coordinates a nickel ion, which itself coordinates an additional chloride ion. Unlike the other chloride ions in the structure, this is not coordinated by polar residues, but buried in the hydrophobic core of the GCN4 adaptor.

The retrospective analysis of the degNHCC construct showed that the redesign improved the confidence of the AlphaFold models and suggests that our design goal to improve structural specificity and stability was achieved. AlphaFold3 models for degNHCC lacking GCN4 adaptors produced higher‐confidence models than for NHCC, especially when including ions (pLDDT >90). When modeled with GCN4 adaptors, the degNHCC construct produced models that closely matched the experimental structure (RMSD <1.5 Å, pLDDT ≈90). The generally confident AlphaFold models for degNHCC without GCN4 adaptors contrast with the IUPRED3 disorder prediction, which is clearly higher for the five designed heptads (Figure 6), and as high as for NHCC.

### 3.4 The role of adaptors in degNHCC

Since degNHCC has much better properties than our original design, but nevertheless is not folded without terminal adaptors, we explored ways to reduce, replace, or entirely eliminate them.

Thus, we shortened the GCN4‐pII adaptors to two heptads (Table S1) and fused these either to both ends of degNHCC or just to the C‐terminus. Only the variant with adaptors at both ends (degNHCC‐(GCN4‐pII)_2hep_NC_) folded, but showed a significantly reduced structural stability compared to degNHCC‐(GCN4‐pII)_2_ (Figure S8). We could nevertheless obtain a crystal structure for it, which agreed with that of degNHCC‐(GCN4‐pII)_2_ to within 0.42 Å for all atoms excluding the GCN4 adaptors. In the construct carrying the C‐terminal GCN4‐pII adaptor, the degNHCC part remained unstructured. Similarly, using the T4 fibritin foldon as a C‐terminal adaptor also failed to yield folded degNHCC. The foldon is a stable, fast‐folding trimeric β‐fold occurring C‐terminally to the central trimeric coiled coil of T4 fibritin. It has been previously used in artificial trimerization approaches (Meier et al., 2004; Wang et al., 2017), but was not successful in this project. Attempts to incorporate cysteine‐containing repeats into degNHCC‐derived peptides were also unsuccessful. These repeats were designed to promote the folding of degNHCC through the formation of specific disulfide bridges similar to those found in the surface protein Tetrabrachion from the hyperthermophilic archaeon *Staphylothermus marinus* (Peters et al., 1996) and in the coiled‐coil domain of cartilage matrix proteins (Dames et al., 1998; Guo et al., 1998), respectively.

Although we did not systematically test the possible adaptors in all combinations and positions in the constructs, we think the results support the conclusion that degNHCC needs adaptors at both ends in order to fold and that otherwise, only the adaptor folds and the designed insert prefers an energetically more favorable, fully solvated but unstructured conformation.

## 4 CONCLUSIONS

In this work, we explore how far the hydrophobic core of a coiled coil can be replaced with polar residues and still yield folded structures. We started by introducing polar core layers into GCN4 variants, but these constructs were not stably folded in solution. We then pursued a de novo design strategy and generated coiled coils in which extended stretches of hydrophobic core residues were replaced by polar interactions. Our first design, NHCC, showed low structural specificity, resulting in dynamic behavior in solution, whereas a redesigned version, degNHCC, formed a more regular and stable trimeric coiled coil. Both NHCC and degNHCC, however, required terminal GCN4 adaptors for stable folding.

Our results show that a considerable length of coiled‐coil sequence can adopt a defined structure without any hydrophobic contacts. In our constructs, this length was 34 residues, but we did not attempt to extend this further and anticipate that possible lengths could be higher, possibly substantially so. Adopting a folded structure appears to require some degree of sequence periodicity, even if degenerate, together with well‐folded flanking adaptors, but these sequences are nevertheless scored as intrinsically unstructured by disorder predictors. This raises questions, particularly in eukaryotic proteomes, about the folding status of such proteins, which are currently annotated as intrinsically disordered proteins (IDPs) in most databases.

The presence of adaptors at both ends of our constructs is clearly imparting stability and structural specificity. As we have observed in many coiled‐coil constructs over two decades, however, the adaptors are not sufficient to fold the entire construct, even when the insert has a sequence periodicity compatible with coiled‐coil structure, and result in two folded adaptors separated by an unstructured segment. Indeed, in NHCC‐(GCN4‐pII)_2_, the central segment remained highly dynamic despite the folded adaptors and was highly sensitive to protease degradation even at room temperature. DegNHCC‐(GCN4‐pII)_2_ behaved more cooperatively and was more resistant to proteolysis, but unfolding experiments still suggest that the main stabilizing contribution comes from the GCN4 adaptors. We therefore interpret the designed regions as sequences that can adopt ordered coiled‐coil conformations when constrained appropriately, rather than as independently folding units. Indeed, despite our redesign efforts, we were unable to produce a coiled coil completely devoid of hydrophobic contacts, able to fold autonomously in solution.

It is possible that completely removing hydrophobic residues may impose too large of an energetic penalty for self‐assembly, even in the presence of an extensive network of polar interactions, suggesting that our results may reflect a fundamental thermodynamic limitation rather than a specific failure in the design strategy. Nevertheless, we achieved greater structural specificity and stability through redesign of our starting construct, indicating that further progress toward a fully polar coiled coil might be possible with current computational approaches.

## AUTHOR CONTRIBUTIONS

**Marcus D. Hartmann:** Conceptualization; investigation; methodology; writing – review and editing; formal analysis; writing – original draft. **Birte Hernandez Alvarez:** Conceptualization; investigation; methodology; writing – review and editing; formal analysis; writing – original draft; visualization. **Mikel Martinez‐Goikoetxea:** Investigation; writing – original draft; writing – review and editing; visualization; methodology; formal analysis. **Andrei N. Lupas:** Conceptualization; investigation; funding acquisition; writing – original draft; methodology; writing – review and editing; formal analysis; supervision; project administration; visualization.

## Supporting information


**Data S1.** Supporting Information.

## Data Availability

The data that supports the findings of this study are available in Supporting Information S1 of this article.
