# Supplementary material for: Building coiled coils with polar cores
Source: Protein Sci. 2026 Sep 16;35(10):e70807. doi: 10.1002/pro.70807 (PMC13580157; doi:10.1002/pro.70807)
Supplement: Supplementary file 1 [file PRO-35-e70807-s001.docx]

**Building coiled coils with polar cores**

Marcus D. Hartmann†, Birte Hernandez Alvarez†, Mikel Martinez-Goikoetxea, and Andrei N. Lupas*

Department of Protein Evolution, Max Planck Institute for Biology Tübingen, 72076 Tübingen, Germany

† joint first authors

* To whom correspondence should be addressed:

Andrei N. Lupas

Tel.: +49 7071 601 341

Fax: +49 7071 601 349

E-mail: andrei.lupas@tuebingen.mpg.de

**Figure S1. AlphaFold3 models of constructs featuring grafted NxxQD motifs in a GCN4-p1 background.** (A) Top and side view of the models, color-coded by pLDDT (low-red to high-blue). The N@a and Q@d residues which coordinate the calcium ion (green sphere) are shown as sticks. (B) Annotated sequence alignment of the wild-type sequence and the designed constructs.

**Figure S2. AlphaFold3 models of constructs featuring grafted NxxQD motifs in a GCN4-p1 background.** (A) Top and side view of the models, color-coded by pLDDT (low-red to high-blue). The N@a and Q@d residues which coordinate the calcium ions (green spheres) are shown as sticks. The model for GCN4-NQD-hep234 is of high confidence without ions (not shown), but drops to medium confidence when calcium ions are added, and only two of the three NxxQD motifs coordinate an ion. We attribute this to quirks in the AlphaFold3 modeling process, as this does not happen with potassium or magnesium ions, which bracket the calcium ion in effective radius, and which are modelled to bind at all three NxxQD motifs. (B) Annotated sequence alignment of the wild-type sequence and the designed constructs.

**Figure S3. Annotated sequences of some salient coiled-coil proteins with polar residues in their core.**

>ACU48812.1 hypothetical protein BMI_I1862 [Brucella microti CCM 4915]

– signal sequence

– putatively unstructured

– coiled coil (hydrophobic and polar core residues)

– anchor domain

MSFFKKNISITAMGGLMLSLAVDAAKAEENVSQVKLPPVFVFELVENQGLANIALIRPRVIAPDNNLRPGG

---IVSG

IAGLLTL

GQENRNL

ISENRQV

INNNTTA

IGQNRTSISTNAKGVADNKAA

IGRNSGRIDTNAKGVADNKTA

IGQNSGRIDTNAKGVADNKTA

IGQNSGRIDANAKGVADNKTA

IGQNSGRIDTNAKGVADNKTA

IGQNSGRIDTNAKGVADNKTA

IGQNSVRIDANAKGVADNKTA

IGRNSGRIDTNAKGVADNRAA

IGRNSGRIDTNAKGVADNRAA

IGRNSGRIDTNAKGVADNRAA

ISQNRGRINANAAGVASNRAA

IRQNSAA

ISALGQR

VDGLQGQ

INSARKEARAGAANAAALSGLRYDNRPGKVSIATGVGGFKGSTALAAGIGYTSKNENARYNVSVAYNEAGTSWNAGASFTLN

>WP_243578192.1 YadA-like family protein [Aggregatibacter segnis ATCC 33393]

– signal sequence

– coiled coil (hydrophobic and polar core residues)

– anchor domain

MKNATFKSSLIALFVAGMCVGTVTAAT

VDDLDKI

VTQHGKV

IGSHEKV

IQAHEKV

IQDHEKV

IQVHEKV

VNKHSDQLADQNDRLVKYKREIDGNKATIDKHDGIITAHENVIQTHEGV-------LKTHEGAIQTHEKAIQTHEQTIQTHEGV

INGHSDQLADHNDRLVKYKREIDDNKAKIDKRDGIITAHENVIQTHEGVLKTHEGAIQTHEGAIQTHEKAIQTHEKAIQTHEGV

INGHSDQLADHNDRLVKYKREIDDNKAKIDKRDGIITAHENVIQAHEGVLKTHEGAIQAHEGAIQAHEKTIQTHEKAIQTHEGV

LKTHEGA

IQTHEKA

INAHKEV

LNTHEGA

INAHREV

LNTHEGA

INAHKAV

LNIHETA

IRENRAN

IASLNTR

VDRLDREVKNGLSTQSALNGLFQPYNVGKLNVSAAVGGYRDKTALAVGTGFRFNEKFAMKAGVAVNTNGGGGMAYNVGANFEF

>ACH84509.1 Haemagluttinin domain protein Lferr_2308 [Acidithiobacillus ferrooxidans ATCC 53993]

– signal sequence

– YadA-like head repeats

– neck

– coiled coil (hydrophobic and polar core residues)

– discontinuities in the heptad pattern; AlphaFold3 models the first as a crossover point in the helical bundle (but not a beta-layer) and the second as a decad (i.e. a heptad ending in a single turn of a 3_10_ helix)

– GIN-type connector

– anchor

MSTFRKKILFVAVSGTFAISMAGGGTADASTVQGGVGLVAALTQ

TLGLGSAVTQYGDSN

NIASGPGSAAGTN

DTAVGVNATSTGTN

SVALGYNSSDGGQNN

VVAVGSATQQRK

IINVAPGTLSQTSTDAVNGSQ

LYATDQ-------QQLTNTSNISNLQNQQKIDQTNISHLQSTVSNISN----LTSVAGDLTAIKQQQQTDMSN-------

IAVNTSDISNLKGQQGTDVTNISNLQKQQATDVSNIANNTSN-------------------------------IASNTSN

IAVNTSDISNLKGQQGTDVTNISNLQKQQATDVSNIANNTSNISNLSNVVGGLTSTAVDLTKIKKQQATDVTNIASNTSN

IASNTSDISNLKNQQGTDVTNISNLQKQQATDVSNIAGNTTN--------------------------------------

IASNTSDISNLKNQQGTDVTNISNLQKQQATDVSNIAGNTTN--------------------------------------

IASNTSDISNLKNQQGTDVTN-----------------------------------------------------------

IASNTKDIKNIKTQQATDVSN--------------IASNTTN--------------------------------------

IASNTSDISNLKTQQGTDVTN-----------------------------------------------------------

IASNTNDIKNVKTQQATDVSN----------------------------------------------------IAMNTSN

ISQLQTIVNGKVATCQVVNG**GLQ**CTY

-------AQAKGTN

DVAAGNGALANGTS

SIAIGTNATATYNG

AVAIGDGARAVADP

ATAIGANAQANANN

STAIGANSTANGIN

SVALGQGSTANRAN

SVSVGNASTGLTRQ

ITNVAPGTTPNDVATVGQ

LQGAVGQ

AQHYAAQVGSVNAAALNAAASAASGQGPNTVAGGYGEYDGQSAFAFTYQHRFNCNWQALLTVGSNGSGKNTEVGAGASYSW

>AAZ76520.1 STEC autoagglutinating adhesin (plasmid) [Escherichia coli]

– signal sequence

– EibA-like head

– YadA-like head repeats

– neck

– coiled coil (hydrophobic and polar core residues)

– anchor

MLKRNCVHSILALTFMATSTHAAPHNGVTYESLEKGKFSDLSSFLKTINLYDKDNNLIDNGKQLDISKAE

NKYLFYTLKTNKQAGLSPEGFGTTLSSIATYYDYAKEQAYFVDKESKKILLSSDGLGNVMVIGPDGYGYT

KHIPEISEYLYKLKINGPLGNAVDMELSGEDIKAIYSEMSYLGSG

KTEAGGRASATGVD

STSLGYNSIANHSN

SIALGANSVTTRSN

EVSIGTKNGETRI

IGGVSDGKLTTDAATVGQLYH

----------EYSIEKVARIEGDAQTLKSANTYTNKQ

VNALEQNTNQQLQHEATARIEGDAQTLKSANTYTNKQ

VNALEQNTNQQLQHEATARIEGDAQTLKSANTYTNKQ

VNALEQNTNQQLQHEATARIEGDAQTLKSANTYTNKQ

VNALEQNTNQQLQHEATARIEGDAQTLKSANTYTNKQ

VNALEQNTNQQ

FRQLRDQ

IIKNRKRSDAGIAGAMAMTAIPMIDGKQYSFGMAASNYRDEQAIAAGIIFRTSENTVVRLNTSWDTQHGTGVATGMSIGW

**Figure S4. samCC analyses of the NHCC experimental structure and the AlphaFold3 models.** From top to bottom, p (helical structural periodicity), P (helical bundle effective periodicity), radius, and axial orientation of the helices (Crick angle).

**Figure S5. Detail of the asymmetry in the N@d layers of the NHCC experimental structure and the AlphaFold3 models.** (Top) Stick representation of the corresponding heptad, with the asymmetric side-chains highlighted (crystal structure in gray, AlphaFold model in cyan). (Bottom) χ1 dihedral angle plots, showing that the asymmetries are not present in the AlphaFold3 model.

**Figure S6. samCC analyses of the degNHCC experimental structure and the AlphaFold3 models.** From top to bottom, p (helical structural periodicity), P (helical bundle effective periodicity), radius, and axial orientation of the helices (Crick angle).

**Figure S7. Detail of the symmetry of the *d* layers of the degNHCC experimental structure and the AlphaFold3 model.** (Top) Stick representation of the corresponding heptad, with the asymmetric side-chains highlighted (crystal structure in gray, AlphaFold model in cyan). (Bottom) χ1 dihedral angle plots, showing that the asymmetries are not present in the AlphaFold3 model.

**Figure. S8 Stability analysis of degNHCC-GCN4-pII variants.** (A) Single CD spectra and (B) thermal melting curves of degNHCC-(GCN4-pII)_2_, degNHCC-(GCN4-pII)_2hep_CN_ and degNHCC-(GCN4-pII)_2hep_C_. The melting curves were recorded at 222 nm and melting temperatures (Tm) are indicated for each protein. (C) Proteinase K digestion assay for degNHCC-(GCN4-pII)_2hep_CN_ and degNHCC-(GCN4-pII)_2hep_C_.

**Table S1. Amino acid sequence of peptides and constructs.**

| Peptide/ construct | Amino acid sequence |
| --- | --- |
| NHCC | *a d a d a d a d a d*  Ac-TDENTKRTDENTKRTDENTKRTDENTKRTDENTKR |
| degNHCC | *a d a d a d a d a d*  Ac-TQENTKRTDENTKHTQETNKRTDQTEQKTQENTKQ |
| NHCC-(GCN4-pII)_2_ | *a d a d a d a d*  MKQIEDKIEEILSKIYHIENEIARIKKL  IDENTKRTDENTKRTDENTKRTDENTKR  MKQIEDKIEEILSKIYHIENEIARIKKLIKLHHHHHH |
| degNHCC-(GCN4-pII)_2_ | *a d a d a d a d a d*  MKQIEDKIEEILSKIYHIENEIARIKKL  IQENTKRTDENTKHTQETNKRTDQTEQKTQENTKQ  MKQIEDKIEEILSKIYHIENEIARIKKLIKLHHHHHH |
| degNHCC-(GCN4-pII)_2hep_NC_ | *a d a d a d a d a d*  MKQIEWKIEEILSK  IQENTKRTDENTKHTQETNKRTDQTEQKTQENTKQ  IYHIENEIARIKKLIKLHHHHHH |
| degNHCC-(GCN4-pII)_2hep_C_ | *a d a d a d a d a d*  MQENTKRTDENTKHTQETNKRTDQTEQKTQENTKQ  IYHIENEIARIKKLIWLHHHHHH |

(Ac-) indicates an acetylated N-terminus.

Coiled coil core positions *a* and *d* are labeled in gray.

Adaptors derived from the GCN4-pII sequence are shown in blue.

Repeats with polar core residues are highlighted in red.

The C-terminal His_6_ linker is shown in black text.

Tryptophan residues inserted for UV-detection during protein purification are underlined.

**Table S2. Crystallographic Data Collection and Refinement Statistics.**

| Structure | **NQD-hep3** w/ calcium | **NQD-hep3** w/o calcium | **NHCC** | **degNHCC** | **degNHCC-(GCN4-pII)_2hep_NC_** |
| --- | --- | --- | --- | --- | --- |
| **Data collection** |  |  |  |  |  |
| Beamline | SLS/X10SA | SLS/X06DA | SLS/X10SA | SLS/X10SA | SLS/X10SA |
| Detector | MarCCD225 | MarCCD225 | MarCCD225 | MarCCD225 | EIGER2 16M |
| Wavelength (*Å)* | 1.2795 | 1.7000 | 0.9764 | 0.9772 | 0.9999 |
| Space group | P321 | C222_1_ | C2 | H32 | P2_1_ |
| Cell parameters  a, b, c, (*Å); ()* | 34.31, 34.31, 48.18; 90.00 | 38.51, 49.77, 62.84; 90.00 | 66.97, 38.62, 165.36; 97.71 | 36.53, 36.53, 561.87; 90.00 | 44.21, 42.99, 58.70; 96.67 |
| Resolution limits (*Å)^a^* | 25.29 – 1.60 (1.69 – 1.60) | 31.42 – 1.89 (2.00 – 1.89) | 33.41 – 1.90 (2.01 – 1.90) | 29.43 – 1.84 (1.95 – 1.84) | 30.72 – 1.77 (1.88 – 1.77) |
| Unique reflections | 8361 (1332) | 9378 (1488) | 33151 (5239) | 12919 (1940) | 40187 (6313) |
| Completeness (%) | 99.3 (99.0) | 99.5 (96.9) | 98.6 (97.1) | 95.9 (92.6) | 96.4 (94.5) |
| Redundancy | 5.75 (5.40) | 7.16 (6.17) | 4.14 (4.13) | 10.50 (10.63) | 3.51 (3.55) |
| I/σI | 17.10 (2.93) | 12.96 (1.76) | 13.25 (1.56) | 15.15 (2.01) | 8.18 (1.07) |
| R_merge_ (%) | 6.4 (92.2) | 8.8 (79.7) | 5.7 (106.9) | 7.9 (159.3) | 7.9 (106.2) |
| CC (1/2) | 99.9 (82.6) | 99.5 (81.9) | 99.9 (85.7) | 99.9 (88.7) | 99.6 (64.7) |
| **Refinement** |  |  |  |  |  |
| Resolution limits (*Å)* | 25.29 – 1.60 | 31.42 – 1.95 | 33.41 – 1.90 | 29.43 – 1.85 | 30.72 – 1.77 |
| R_cryst_ (%) | 17.0 | 25.7 | 25.3 | 22.8 | 26.1 |
| R_free_ (%) | 20.4 | 29.1 | 28.7 | 27.2 | 29.0 |
| Mean B value (*Å^2^)* | 23.0 | 37.1 | 45.6 | 52.9 | 37.9 |
| Ramachandran preferred/allowed/outlier regions (%) | 100.0/0.0/0.0 | 98.1/1.9/0.0 | 99.6/0.4/0.0 | 98.9/1.1/0.0 | 100.0/0.0/0.0 |
| **PDB accession code** | **30LG** | **30LH** | **30LI** | **30LL** | **30YL** |

^a^ Values in parenthesis refer to the highest-resolution shell.

**Table S3. Summary of survey of sequences featuring NxxQD motifs within predicted coiled-coil regions.**

| Number of sequences | Filtering step | Percent w.r.t total | Percent w.r.t previous step |
| --- | --- | --- | --- |
| 429,368,622 | AlphaFold_UniProt100 (2024) | 100.0% | 0.0% |
| 3,970,251 | Seqs. with NxxQD motif | 0.9% | 0.9% |
| 139,429 | Seqs. with >50% COILS prob. | 0.0315% | 3.5% |
| 124,994 | Non-identical | 0.0284% | 90.0% |

**Table S4. Tallies of the 7 most common predicted registers for NxxQD in natural coiled-coil sequences.** The intended register of the NxxQD motif in our designed construct was *abcdef*.

| Motif register NxxQD | Counts | Percent |
| --- | --- | --- |
| bcdef | 47126 | 33.79% |
| fgabc | 42853 | 30.73% |
| cdefg | 32756 | 23.49% |
| abcde | 5423 | 3.89% |
| efgab | 4695 | 3.37% |
| gabcd | 3321 | 2.37% |
| gabcg | 1033 | 0.74% |
| ... | ... | ... |
| Total | 139429 | 100% |
